# Complete Genome Sequence of Stenotrophomonas Phage Pokken

**DOI:** 10.1128/MRA.01095-19

**Published:** 2019-10-17

**Authors:** Ashley Hayden, Nicholas Martinez, Russell Moreland, Mei Liu, Carlos F. Gonzalez, Jason J. Gill, Jolene Ramsey

**Affiliations:** a Center for Phage Technology, Texas A&M University, College Station, Texas, USA; b Department of Biology, Texas A&M University, College Station, Texas, USA; Loyola University Chicago

## Abstract

Stenotrophomonas maltophilia is a Gram-negative bacterium associated with multidrug-resistant nosocomial infections, a problem for immunocompromised patients and those with cystic fibrosis. Here, we present the new S. maltophilia-infecting podophage Pokken. Its 76,239-bp genome, with 92 protein-coding genes and 5 tRNA genes predicted, is similar to that of phage N4.

## ANNOUNCEMENT

Stenotrophomonas maltophilia is an emerging Gram-negative multidrug-resistant opportunistic pathogen (1). Increasingly, S. maltophilia has been seen in nosocomial infections in intensive care units and in immunocompromised individuals (2). Additionally, S. maltophilia is associated with severe pulmonary disease in cystic fibrosis patients (3). In the interest of exploring potential therapeutic treatment options, we isolated and annotated the genome of S. maltophilia podophage Pokken.

Pokken was isolated from filtered (filter size, 0.2 μm) freshwater collected at Camp Creek Lake (Franklin, TX) and propagated aerobically on S. maltophilia (ATCC 17807) at 30°C in nutrient broth or agar (BD) with the soft-agar overlay method (4). To determine phage morphology, samples were negatively stained with 2% (wt/vol) uranyl acetate and viewed by transmission electron microscopy at the Texas A&M Microscopy and Imaging Center (5). DNA was purified with the modified Promega Wizard DNA clean-up system shotgun library preparation protocol, prepared as Illumina TruSeq libraries with a Nano low-throughput kit, and sequenced on an Illumina MiSeq instrument with paired-end 250-bp reads using v2 500-cycle chemistry (6). The 414,121 total reads in the phage-containing index were quality controlled with FastQC (www.bioinformatics.babraham.ac.uk/projects/fastqc) and trimmed using the FastX Toolkit v0.0.14 (http://hannonlab.cshl.edu/fastx_toolkit/). With SPAdes v3.5.0 at the default settings, a raw contig at 188.1-fold coverage was assembled (7). To verify that the complete sequence was present, PCR products amplified off the contig ends (forward, 5′-GGGTACATCCCGAGTAAGAAAC-3′; reverse, 5′-GTGACCTCCATGGTTCGATAG-3′) were sequenced by the Sanger method. Protein-coding genes were annotated by using GLIMMER v3.0 and MetaGeneAnnotator v1.0 (8, 9). tRNA genes were detected with ARAGORN v2.36 (10). TransTermHP v2.09 analysis was used to annotate termination sites (rho independent) (11). Putative gene functions were assigned based on conserved protein domains, which were detected using InterProScan v5.33-72 and similarity search results from BLAST v2.2.31 against the following databases, with a 0.001 maximum expectation value cutoff: NCBI nonredundant, UniProtKB Swiss-Prot, and TrEMBL (12–14). Potential transmembrane domains were detected with TMHMM v2.0 (15). Genome-wide DNA sequence similarity between Pokken and other phages was calculated by progressiveMauve v2.4.0 (16). Genomic terminus type was predicted by PhageTerm (17). All tools were accessed at the Center for Phage Technology Galaxy interface, and Web Apollo was used for annotation (https://cpt.tamu.edu/galaxy-pub/) (18, 19). Unless otherwise stated, all tools were executed using default parameters.

The 76,239-bp genome of podophage Pokken has a 55% G+C content, lower than the 66.8% average G+C content of the host (20). Our analysis predicted 92 protein-coding genes and 5 tRNA genes, yielding an overall 92.8% coding density. Of the 29 protein-coding genes that were assigned putative functions, 18 were similar by BLASTp search to enterobacterial phage N4 (GenBank accession number NC_008720). Pokken has an overall 29.94% identity with phage N4 and was predicted to contain 627-bp direct terminal repeats, which were somewhat longer than the direct terminal repeats in phage N4 (21). Additionally, Pokken encodes four putative tail fiber proteins in a row (NCBI accession number QEG09305 to QEG09308), and bacteriophage Prado encodes four tail fiber proteins in a row similar to those of Pokken (GenBank accession number KF626667) (22).

### Data availability.

The genome sequence and associated data for phage Pokken were deposited under GenBank accession number MN062186, BioProject accession number PRJNA222858, SRA accession number SRR8892199, and BioSample accession number SAMN11411460.
